# Health belief model based predictors and perceptions of COVID-19 vaccination amongst frontline health workers

**DOI:** 10.6026/973206300211404

**Published:** 2025-06-30

**Authors:** Jitendra Majhi, Paramita Sengupta, Preetika Banerjee, Aloke Biswas, Kayur Mehta, Anita Shet, Ninad Vilas Nagrale

**Affiliations:** 1 Department of Community Medicine & Family Medicine, All India Institute of Medical Sciences, Kalyani, West Bengal, India; 2 International Vaccine Access Center, Johns Hopkins Bloomberg School of Public Health, Baltimore, Maryland, USA; 3 International Health, Johns Hopkins Bloomberg School of Public Health, Baltimore, Maryland, USA; 4 Department of Forensic Medicine & Toxicology, All India Institute of Medical Sciences, Kalyani, West Bengal, India

**Keywords:** COVID-19, vaccine acceptance, health belief model, ordinal regression, ASHAs

## Abstract

COVID-19 pandemic presented with a scenario wherein the healthcare delivery system was overwhelmed by unprecedented COVID morbidities. Vaccines were made available in a short span of time during the pandemic for the public that required the frontline workers especially the ASHAs to overcome their own hesitancies of the newer vaccine before convincing the community for vaccination. A cross-sectional survey on the perceptions of COVID vaccination was conducted wherein 74 ASHAs participated in the interview involving Likert scale responses on vaccine acceptance which were modelled on Health Belief Model (HBM) to find predictors of vaccine acceptance by performing ordinal regression analysis. It was seen that the domains of 'Benefit', 'Barrier' and 'Modifying Variable' appeared to increase the acceptance of the vaccine whereas the domains of 'Susceptibility' and 'Severity' had decreasing effect on acceptance, of which only 'Severity' domain significantly had a negative effect on COVID vaccine acceptance amongst the frontline workers. HBM is a useful tool which can provide information about the facilitating and negating factors of newer vaccines introduction which can be utilized by public health researchers to better understand the perceptions and accordingly vaccine introduction approaches can be modified.

## Background:

The COVID-19 pandemic had an unprecedented catastrophic impact upon mankind in the recent times. The pandemic caused an overwhelming burden upon the government health care facilities and posed a daunting task for the healthcare providers in catering to the loads of COVID-19 cases coming to their setup, in times when the standalone local private practitioners had to shut down due to health concerns of their own [1]. The role of Frontline Workers in carrying out the healthcare services during the pandemic spanned not only in the community but also from door to door to sustain the health programs especially mother and child health programs including immunization. Righteously, the ASHAs' (Accredited Social Health Activists) contribution in India has been recognized and have been awarded by the WHO as Global Health Leaders in 2022 [2]. COVID-19 vaccination in India also experienced similar apprehensions as with other newly introduced vaccines [3], but the communities coming to the terms of acceptance was relatively shorter due to multiple large scale campaigns from the highest of the offices of the Government [4]. The perception of Frontline workers especially ASHAs of District Nadia, West Bengal, overcoming their apprehensions about COVID-19 vaccination and motivate others in the community where they serve to accept the new vaccine is relevant. Therefore, it is of the interest to describe the various domains that explain the behaviours of frontline workers that play a role to contribute towards vaccine acceptance, and the attributes of such behaviours may help researchers and public health experts to consider them when faced with similar overwhelming situation requiring immediate public health preventive actions in the future.

## Materials and Methods:

Frontline Workers working as ASHAs under National Health Mission (NHM) in the Rural and Urban Field Practice Areas of the Department of Community Medicine & Family Medicine, AIIMS Kalyani were approached and interviewed regarding their opinions about the COVID-19 Vaccine, vaccination programme and acceptance of the vaccine personally and in the communities where they serve during the period September 2021 To February 2022. The Health Belief Model (HBM) is a theoretical model which is often used to explain individual changes in health behaviours. HBM is one of the most widely used models for understanding health behaviours. Components of the HBM focuses on individual beliefs about health conditions, which determine individual health related behaviours. The model defines the key factors that influence health behaviours as an individual's perceived threat to sickness or disease (perceived susceptibility), belief of consequence (perceived severity), potential positive benefits of action (perceived benefits), perceived barriers to action, exposure to factors that prompt action (cues to action), and confidence in ability to succeed (self-efficacy) [5]. The interview tool was a questionnaire which was used to capture data pertaining to COVID-19 vaccination containing sections (7 in number) on general information, COVID-19 personal experience, COVID-19 safety precautions, side-effects associated with vaccination, COVID-19 Vaccine perceptions, COVID-19 Vaccine preparedness and broader family and community acceptance. The questionnaire was developed by researchers at the Johns Hopkins Maternal and Child Health Center in India and Department of Community Medicine & Family Medicine, AIIMS Kalyani using vaccine hesitancy questionnaire guidelines developed by the Strategic Advisory Group of Experts on Immunization (SAGE) Working Group of the World Health Organization (WHO) [6]. The survey also used components similar to the Kaiser Family Foundation (KFF) survey done in the United States to assess perceptions of the COVID-19 vaccination [7]. The questions and domains were aligned with the objectives of capturing cross-sectional perceptions on vaccine acceptance from HCWs at the time of vaccine rollout within the Indian context. Participants were asked about their decision to take COVID-19 vaccine by asking them 10 questions whose response was recorded in the Likert Scale about their perceptions on COVID-19 Vaccination on five domains of the Health Belief Model explored by two questions in each of the domains, viz. first, the domain of perceived susceptibility whether there is any perceived harm in taking the vaccine, and whether post vaccination there is need to wear masks or practice social distancing; second, domain of the perceived severity whether the vaccine might cause serious side effects and whether the recipient may have some unforeseen side effects in the future; third, domain of the perceived benefits whether the vaccine will be useful in protecting from COVID infection and whether the benefits of vaccination outweighs the risks involved; fourth, domain of the perceived barriers whether the vaccine may be faulty or fake and whether the vaccine has been hastily developed and approved; fifth, domain of the perceived modifying variables whether the vaccine is being promoted for commercial gains of pharmaceutical companies and whether the vaccine is available free of cost. These five domains of the health belief models explored by two questions in each domain were treated as the predictors of vaccine acceptance which was in turn were also explored by two separate questions in Likert Scale which were, whether the recipient can contract COVID infection because of the virtue of working as a frontline worker and whether the members in their communities would accept COVID-19 vaccination. Perception of COVID-19 vaccination were captured using questions that were designed in a Likert Scale moulded within the framework of the Health Belief Model to identify the factors that motivated the ASHAs for vaccination for themselves and motivating others in the community for acceptance either positively or otherwise.

## Results:

An ordinal logistic regression analysis based on the Health Belief Model which was performed in SPSS software, was used to analyze the data. The current study explores the predictors of vaccine acceptance amongst the frontline workers comprising of ASHAs across both the rural and urban field practice areas of AIIMS Kalyani located in rural Chakdaha Block & urban Kalyani Municipality respectively. We enrolled 74 ASHAs in the study. Most (51.3%) of the study participants belonged to middle aged group (36 to 45 years) with a mean (±SD) age of 38.4 (8.7) years. Most (66.2%) of the study participants were providing community healthcare services in rural area (Table 1 - see PDF). The descriptive details of the ASHA's characteristics of prevailing practices during COVID-19 scenario are summarized in (Table 2 - see PDF). Most of the respondents (94.6%) were vaccinated with two doses of Covishield vaccine. Vaccine was not refused amongst 97.3% of the participants. Only a few (5.45%) were hesitant of getting infected by COVID-19 virus during their duty hours. While majority (70.3%) of them never had to undergo any testing for COVID-19. Out of the 22 respondents who got tested for COVID-19 at least once, 03 (13.6%) tested positive for COVID (Figure 1 - see PDF). Majority (90.3%) of the study participants perceived that they are 'very likely' of getting infected by COVID-19 disease, though only one third (32.4%) of them agreed of maintaining 6 feet social distancing at work place. All ASHAs had been trained on COVID-19 preventive measures and precautions while one quarter (24.0%) of them revealed that the training was not sufficient. More than one third (37.9%) of them opined that sufficient stock of PPE was not in place. Almost all (94.5%) of them agreed that adequate information was available to public about COVID-19 vaccines. More than half (58.1%) of the study participants felt free to ask supervisors or government officials about COVID-19 vaccines. Almost all (97.3%) of them agreed to the need of a plan for rapid provision of COVID-19 vaccines by district and supervisors to the Front Line Workers and Community Health Workers, while all of them agreed that such a plan should be there for the community (Table 2 - see PDF, Figure 2 - see PDF).

Around 24.3% of the ASHAs felt that community's refusal to COVID-19 vaccination was due to the extra time taken off from their work for COVID-19 vaccination while only few (4.1%) of them thought that communities' refusal's to COVID-19 vaccination was due to the side effects of vaccines (Table 2 - see PDF, 3 - see PDF & Figure 3 - see PDF). Nearly all of them felt that the COVID-19 vaccine as the best choice to end the pandemic (Table 2 - see PDF & 4 - see PDF, Figure 4 - see PDF). Majority (70.3%) of them felt that service area has sufficient staff to roll out COVID-19 vaccination to the public whereas, 90.5% of the participants felt that service area has adequate supplies needed to roll out and store the COVID-19 vaccination. No participant felt that COVID-19 vaccine roll out decreased the number of routine vaccines under NIS (Table 2 - see PDF, 5 - see PDF, Figure 5 - see PDF). Nearly all the participants (98.6%) revealed that their family members were vaccinated against Covid-19 disease, with no difficulty faced in accessing the vaccine. Of those getting vaccinated, 86.5% of their family members were never diagnosed having COVID-19 disease and only one family member was hospitalized after being diagnosed with COVID-19. Majority (89.2%) of the participants opined that the door to door approach was the best mode of awareness campaign for increasing COVID-19 vaccine acceptance by the community members. Most (86.5%) of them thought that the health workers were the best person for endorsement to impact community member's decision for increasing COVID-19 vaccine acceptance (Table 2 - see PDF, 6 - see PDF, Figure 6 - see PDF).

## Modelling:

The specific questions pertaining to behaviours influencing COVID-19 vaccination were grouped into the HBM variables of Perceived Susceptibility, Perceived Severity, Perceived Benefits, Perceived Barriers and Modifying Variables that affected the outcome i.e. dependent variable Vaccine Acceptance (Figure 5 - see PDF). For exploring each variable, two questions were asked for each variable to understand the domains of HBM (Figure 6 - see PDF). Two negatively framed questions viz. PSus_2 and MV_1 were re-framed into positively framed questions by recoding the responses inversely, in agreement with the remaining questions which were all positively framed (Table 3 - see PDF).

## Statistical test for determining the normality of data:

The individual Health Belief Model variables including the dependent variable of vaccine acceptance studied in the research of 74 participants were found to be significant in the Kolmogorov-Smirnov Test implying a non-parametric distribution of the data (Table 4 - see PDF).

## Ordinal regression analysis:

The SPSS ordinal regression procedure or PLUM (Polytomous Universal Model) is an extension of the general linear model to ordinal categorical data [8]. In Table 5 (see PDF) the parameter estimates is depicted which provides information about the domains which were either positively or negatively played their role in deciding the acceptance of vaccine amongst the ASHA workers. The benefit domain, barrier domain and modifying variable domain have positive estimates or coefficients of 0.718, 0.483 and 2.184 respectively. The positive estimates portray that for increase in the values of these domains would increase the acceptance of the vaccine. However, none of these positive domains were found to be significantly associated with vaccine acceptance. The susceptibility domain and severity domain have negative estimates of -1.78 and -2.231 respectively. Negative estimates inform that with the increase of (negative) values would decrease the level of vaccine acceptance amongst the ASHAs. Of these negative estimates, susceptibility was not significant. Only the domain of severity was found to be significantly associated with vaccine acceptance in the ASHAs in this study. Severity domain was a significant predictor of vaccine acceptance. For every one unit decrease in severity there is a predicted decrease of -2.231 in the odds of acceptance of the vaccine. The variable of age group categories had a positive estimate in the youngest age group between 19-28 years, whereas the estimates were negative in the older age groups of 29-38 years (-16.156) and 39-48 years (-18.459). The oldest age group of 49-58 years was considered redundant by the model. However, the age group and locality variables were not significant.

The model fitting information is projected as Chi-Square test value of 16.925 in Table 6 (see PDF) which is found to be statistically significant showing that the model fits the data very well. The Goodness-of-Fit information (Table 7 - see PDF) shows the Pearson and Deviance's Chi-Square values to be non-significant. Non-significant test results are the indications that the model fits the information well, these results suggest a decent model fit and the observed data is consistent with the fitted model. Pseudo R Squared values as depicted in Table 8 (see PDF) are one of the outputs of Ordinal Regression. These values are used when the output of interest is in ordinal scale. Although Pseudo R Squared is not exactly R2 from linear regression yet it still gives a rough idea about the predictive power of the model given all the assumptions are met. Three methods viz. Cox & Snell, McFadden and Nagelkerke have been depicted. However, Negelkerke's method have the largest Pseudo R squared statistics which is the best according to this measure.

## Discussion:

Most of the studies on COVID-19 vaccine acceptance has been done in the online mode [9, 10, 11, 12, 13, 14, 15, 16-17], followed by using social media platforms [18, 19], web based platforms [20], telephone based [21] and email based surveys [22] to name a few. Studies utilizing Health Belief Model (HBM) to explore COVID-19 vaccine acceptance have also been commonly done before. Few studies have been done amongst the Healthcare Workers utilizing the HBM [23, 24, 25, 26]. The current study was conducted after the declaration of the end of pandemic situation in the country, which made it possible to do face to face interview. The survey showed that 97.3% ASHAs had not refused vaccination, 94.6% of the ASHAs had received complete vaccination, 91.9% had no hesitancy of infected by the COVID-19 virus, which shows that ASHAs have overall good confidence based upon their personal experiences. This finding of overall good experience by healthcare workers is similar to another survey in China by Wang *et al.* [26]. About 70.3% never had to undergo COVID tests and those who underwent testing 86.4% tested negative for COVID. Such desirable experience may be due to the waning off of the pandemic and easing of restrictions during the period of the study. The hurdles and hardships faced during the testing times of the pandemic may have boosted the ASHA's level of confidence to face the situation in their communities [27]. 90.3% of ASHAs had feeling of very likely being infected by COVID disease. 86.5% of ASHAs had received training on COVID-19 preventive measures and precautions. 70.2% of ASHAs felt they had been trained sufficiently. 58.1% ASHAs were comfortable to ask their supervisors or officials about their queries on the vaccine. These activities of ASHA's involvement and participation had a satisfactory effect on practising COVID safety precautions such as maintenance of 6 feet social distancing at work place (32.4%) and maintaining sufficient stock of PPE (72.1%) and dissemination of information to the public about the COVID-19 vaccines (94.5%). In addition, the ASHA's participation had also made 97.3% and 100% of ASHA to be involved in development of plans for facilitating rapid provisions of COVID-19 vaccines from the district and supervisors to the frontline workers and to the community respectively. Majority of the community were not concerned with the side effects of the COVID-19 vaccination (87.9%) and the concerns about the non-completion of personal responsibilities were not experienced by the majority of the community (74.3%). Almost 97.3% of ASHAs believed that the vaccine is the best choice to end the pandemic. 70.3% of ASHA believe that the staff are sufficient, 90.5% believe that supplies are adequate. 97.3% did not believe that COVID-19 is hampering the supply of routine vaccines under National Immunization Schedule (NIS).

Therefore, overall there was relatively a positive vaccine perception and satisfactory practices which was also reported in studies conducted amongst healthcare workers in Jeddah, Saudi Arabia by Alfosail *et al.* [28] and in China by Wang *et al.* [26]. Other studies with positive perceptions were mostly conducted using online platforms [9, 10, 20, 29 and 30]. Some studies wherein the perception of acceptance of the vaccine was poor was reported by Getachew *et al.* [24] done by face to face interview amongst health care professionals and by Chen *et al.* [17] done by online mode in general population. A person's subjective estimation of their likelihood of having a health issue is known as perceived susceptibility. According to the Health Belief Model (HBM), Individuals who believe they are vulnerable to a specific health issue will take actions to lower their chance of experiencing that issue. Individuals who don't think they are susceptible to a certain ailment might not believe they are. While some may accept the chance of contracting the sickness, they think it implausible. Individuals who think they have little chance of getting sick are more likely to take risks or participate in dangerous practices. Individuals who believe there is a significant chance they will experience a specific health issue first-hand are more inclined to take steps to lower their risk. Out of the two questions asked in this domain for vaccine acceptance, one had to be reframed positively. To the first question whether" I think there is no harm in taking the COVID vaccine", maximum responses (48) were strongly agreed that there is no harm in taking the vaccine, followed by 24 responses towards agreeing and only 2 responses was neither in the Likert scale. None of the ASHAs disagreed or strongly disagreed to this question. To the second question asked in this domain which required reframing, whether "after taking COVID-19 vaccine, there is need to wear masks or do social distancing" most of the responses were towards strongly agreed followed by 19 responses as agreed. 2 ASHAs strongly disagreed and no responses were registered as disagree or neither in the Likert scale. Overall in this domain most of the responses are indicate of positive behavior except 2 responses in negative behavior. With a P-value of 0.303 and CI of -5.241 to + 1.661, the final regression model also puts this domain to be none significant predictor in determining the vaccine acceptance amongst the frontline workers. However, the estimate is a negative value (-1.78) which implies that the factor has a negative effect on the acceptance of the vaccine. This means that as the values of the independent variable which is "perceived susceptibility" increases, there is a decreased probability of falling at a higher level on the dependent variable which is "vaccine acceptance."

The subjective evaluation of the seriousness of a health issue and its possible out-comes is known as perceived severity. According to the Health Belief Model (HBM), individuals who view a particular health issue as serious are more likely to take actions to stop it from happening or lessen its severity. Views on the illness itself and its wider effects on a person's ability to perform in social and occupational tasks are all included in the concept of perceived seriousness. For example, someone may believe that flu is not a dangerous medical ailment, but if they believe that missing work for many days will have a significant financial impact, they may believe that flu is a particularly critical condition. Furthermore, compared to those who perceived the condition as moderate, those who rated the condition as high were much more likely to have obtained the vaccination. The domain of perceived severity was covered by two questions viz. "... the vaccine might cause serious side effects" and "... I might have some unforeseen future effects of the vaccine". The first question on perceived severity had maximum 86.5% (64) responses as disagree, with remaining responses of strongly disagree to be 6.8% (5 ASHAs), neither to be 5.4% (4 ASHAs), agree to be 4.1% (3 ASHAs) and strongly agree to be 1.4% (1 ASHA). Similarly, the second question on perceived severity had maximum 82.4% (61 ASHAs) response as disagree, followed by 16.2% (12 ASHAs) responses as neither and 1.4% (1 ASHA) response as agree. No responses were recorded as in the either extremes (strongly disagree - 0; strongly agree - 0) of the Likert scale response.

Interestingly, this was the only predictor variable which proved to be significant factor for COVID-19 vaccine acceptance amongst frontline workers when modelled in the regression equation with a P value of 0.033 and CI of -4.284 to -0.179. The estimate is a negative value (-2.231) which implies that the factor has a negative effect on the acceptance of the vaccine. This means that as the values of the independent variable which is "perceived severity" increases, there is a significant decreased probability of falling at a higher level on the dependent variable which is "vaccine acceptance." An individual's evaluation of the worth or effectiveness of adopting a health-promoting action to lower risk of disease is referred to as perceived benefits. Regardless of objective information about the efficacy of a certain action, a person is likely to engage in it if they believe it will lessen their vulnerability to a health problem or lessen the severity of the problem. Perceived rewards of action have an impact on habits connected to health. The domain of perceived benefits was explored by two questions viz. "... I believe COVID vaccine will be useful in protecting me from COVID infection" and "... I believe the benefits of taking the vaccine outweighs the risks involved". To the first question asked in this domain, maximum responses 44.6% (33 ASHAs) were strongly agree, followed by 36.5% (27 ASHAs) responses as agree. 17.6% (13) responses were neither, with only 1.4% (1 ASHA) response as disagree. None of the ASHAs responded as strongly disagree with this question. Similarly, to the second question of this domain registered maximum responses of 58.1% (43 ASHAs) as strongly agree, followed by 31.1% (23 ASHAs) responses as agree. 2.7% (2 ASHAs) responded as neither and 8.1% (6 ASHAs) responded as disagree.

None of the responses were recorded as strongly disagree in this question. However, perceived benefits as a predictor variable were found to be non-significant factor for COVID-19 vaccine acceptance amongst frontline workers when modelled in the regression equation with a P value of 0.306 and CI of -0.656 to 2.091. The estimate of "perceived benefits" is a positive value (0.718) implying that there is an increased probability of vaccine acceptance increases as this independent variable increases. Perceived barriers pertain to an individual's evaluation of the impediments to altering their behavior. Barriers may keep someone from engaging in an activity that promotes health, even if they think it will successfully lessen the threat posed by a health condition and view it as such. Perceived obstacles to action also influence health-related behaviors. In other words, for behavior to change, the perceived advantages must exceed the perceived obstacles. The perceived cost, difficulty, danger, and inconvenience of engaging in the behaviour are some of the perceived barriers that prevent individuals from acting. In the study this domain of vaccine acceptance was explored by the following questions viz. "COVID-19 may be faulty or fake" and "COVID -19 vaccines was hastily developed and approved". To the first question maximum responses 90.5% (67 ASHAs) were recorded as disagree followed by 6.8% (5 ASHAs) responses as strongly disagree. 2.7% (2 ASHAs) responded neither, and none was documented in the Likert scale of agree and strongly agree. Similarly, to the second question, maximum 73% (54) disagreed and 2.7% (2) strongly disagreed. 13.5% (10) agreed, while 4.1% (3) strongly agreed to this statement. 6.8% (5 ASHAs) responded as neither. Eventually, when this predictor variable was modelled in the ordinal regression equation, it turned out to be a non-significant factor in determining the COVID-19 vaccine acceptance amongst the frontline workers with a P value of 0.623 and CI of -1.442 to 2.409. The HBM suggests that modifying variables affect health-related behaviours indirectly by affecting perceived seriousness, susceptibility, benefits, and barriers.

The vaccine acceptance domain of modifying variables was explored by two questions viz. "COVID 19 vaccine is being promoted for commercial gains of pharmaceutical companies" and " COVID 19 vaccine is/was available free of cost". The second question was originally asked in a negative sense which had to be reframed positively in the HBM. The first question was responded maximum 85.1% (63 ASHAs) as agree. 4.1% (3) strongly agreed to this statement. 10.8% (8) responded neither to any of the responses in the Likert scale. No responses were recorded in disagree and strongly disagree Likert. To the second question, maximum 93.2% (69 ASHAs) responded as strongly agreed, followed by 5.4% (4 ASHAs) responding as agree. 1.4% (1 ASHA) responded as disagree. None of the responses were recorded as strongly disagree and neither. However, this predictor variable of modifying variable in the HBM turned out to be non-significant in the ordinal regression model in determining the COVID-19 vaccine acceptance amongst frontline workers. Variables like age groups and localities whether rural or urban were also fitted as independent variables in the regression equation for exploring role in the acceptance of COVID 19 vaccine. It was observed that the youngest age category between the ages of 19-28 years had positive estimate value, as opposed to the other older age groups which had negative estimates. However, this variable of age groups was not statistically significant. On the other hand, the model showed that the rural community had positive acceptance of vaccine as compared to their urban counterparts. But this also could not be ascertained based on the statistical significance criteria.

## Limitations:

In this study the acceptance of COVID-19 vaccine amongst frontline workers was studied by application of health belief model wherein various independent factors were analyzed in the domains within the paradigm of the model like perceived susceptibility, severity, barriers, benefits and modifying variables. These domains were fitted in an ordinal regression model along with confounding co-factors like age and locality. It was seen that out of the five domains explored, the perceived severity domain was significantly associated with COVID-19 vaccine acceptance amongst the frontline workers. Although all the ASHA workers working in the field practice areas of AIIMS Kalyani were interviewed, the sample size of the study may be small to make the vaccine acceptance estimates generalizable to the larger community. The period when the survey was undertaken, the prevailing COVID conditions were mostly waning off and restrictions were lifted off and the conditions were returning back to normalcy. The apprehensions about the vaccine may have lessened and at the same time the general population was more in favour of the vaccines.

## Conclusion:

Health belief model could explain the predictors that play a role in the acceptance of a new vaccine amongst the frontline health workers and in the community by robust modelling parameters of an ordinal regression modelling procedure. Domains like perceived susceptibility and perceived severity were negative factors that decreased the acceptance of the new vaccine, whereas, domains like perceived benefits, perceived barrier and modifying variables were positive factors that increased the acceptance, of which the domain of severity was significantly decreasing the vaccine acceptance amongst ASHAs. Knowledge of the positive and negative factors may be useful for the public health professionals to better understand the approaches to introduce a new vaccine and also the challenges faced to mitigate the fears amongst the consumers for vaccine acceptance. This is not only for COVID Vaccines but also for other newer vaccines that may be introduced in the future.

## Author's contribution:

Conceptualization: JM, PS and PB; Experimental work and analysis: JM, PS and PB; Wrote the manuscript: JM, AB, NN and PS; Reviewed manuscript: JM, PS, PB, AB, KM, AS and NN.

## Financial support:

Extramural funds received for Project MATCHES from the International Institute of Innovation & Technology (I3T), Kolkata.
